# Do We Need Titanium Dioxide (TiO_2_) Nanoparticles in Face Masks?

**DOI:** 10.3390/toxics13040244

**Published:** 2025-03-25

**Authors:** Stijn Everaert, Lode Godderis, Jean-Marie Raquez, Greet Schoeters, Pieter Spanoghe, Jonas Moens, Luc Hens, Olivier Michel, Dirk Adang, Norbert Fraeyman

**Affiliations:** 1 Chemical Environmental Factors Group, Superior Health Council, 1210 Brussels, Belgium; 2 Center for Environment and Health, Department of Public Health and Primary Care, KU Leuven, 3000 Leuven, Belgium; lode.godderis@kuleuven.be; 3 IDEWE, 3001 Heverlee, Belgium; 4 Polymer and Composite Materials Department, University of Mons, 7000 Mons, Belgium; 5 Department of Biomedical Sciences, University of Antwerp, 2610 Antwerp, Belgium; greet.schoeters@uantwerpen.be; 6 Department of Plants and Crops, Ghent University, 9000 Ghent, Belgium; 7 Belgian Poison Centre, 1120 Brussels, Belgium; jonas.moens@poisoncentre.be; 8 Vlaamse Instelling voor Technologisch Onderzoek, 2400 Mol, Belgium; 9 Faculté de Médecine, Université Libre de Bruxelles, 1070 Brussels, Belgium; olivier.michel@ulb.be; 10 Faculty of Medicine and Life Sciences, Hasselt University, 3590 Diepenbeek, Belgium; 11 Environmental Department, Ghent University Hospital, 9000 Ghent, Belgium

**Keywords:** inhalation exposure, TiO_2_, nanoparticles, human health, face masks

## Abstract

The use of face masks has proven to be an effective preventive measure during the COVID-19 pandemic. However, concerns have emerged regarding the safety of metal (nano)particles incorporated into face masks for antimicrobial purposes. Specifically, this review examines the risks associated with TiO_2_ nanoparticles (NPs), which are classified as a possible human carcinogen. The inhalation of TiO_2_ NPs can cause multiple adverse effects, including oxidative stress, pulmonary inflammation, histopathological changes, and (secondary) genotoxicity. Different aspects are discussed, such as the composition and filtration efficiency of face masks, the antimicrobial mode of action and effectiveness of various metals, and the hazards of TiO_2_ NPs to human health, including exposure limits. A conservative risk assessment was conducted using different worst-case scenarios of potential (sub)chronic TiO_2_ exposure, derived from published leaching experiments. Most face masks are considered safe, especially for occasional or single use. However, the nanosafety of a minority of face masks on the European market may be inadequate for prolonged and intensive use. Important uncertainties remain, including the risks of combined exposure to TiO_2_ NPs and silver biocides, and the lack of direct exposure measurements. Considering the potential safety issues and the limited added protective value of TiO_2_ NPs, it is recommended to ban all applications of TiO_2_ in face masks based on the precautionary principle.

## 1. Introduction

Between 2020 and 2021, the WHO estimated 14.83 million global excess deaths associated with the COVID-19 pandemic [1]. The high transmission and mortality rate of the SARS-CoV-2 virus required urgent sanitary and preventive measures to slow down the spread of the infection [2,3,4,5]. Besides social distancing and other measures, the massive use of face masks proved particularly effective in reducing viral transmission [6,7,8,9,10,11,12,13,14,15,16]. In several meta-analyses, wearing masks (surgical masks, respirators, etc.) was associated with a significantly reduced risk of COVID-19 infection: a 51% [12], 62% [13] and 85% [8] reduction in the (adjusted) Odds Ratio (aOR), and an 88% reduction in the Relative Risk (RR) [14].

Given their extensive use and variation in composition, some authorities [17,18,19,20,21] and consumer organisations [22] wanted to investigate the intrinsic safety of face masks. For example, face masks were found to contribute to the personal and environmental burden of nano- and microplastics [23,24,25,26,27,28,29]. In addition, they can contain traces of harmful chemicals, both intentionally added (such as plasticizers, adhesives, solvents, dyes, etc.) and unintentionally present (such as impurities, contaminants, and degradation products). These include amongst others formaldehyde [30,31,32], phthalates [24,25,33,34,35,36,37], organophosphorus esters (OPEs) and organophosphate flame retardants (OPFRs) [36,38,39], per- and polyfluoroalkyl substances (PFAS) [36,40], volatile organic compounds (VOCs) [36,37,39,41,42,43], polycyclic aromatic hydrocarbons (PAHs) [37,39], reactive carbonyl species [37], and metals (e.g., Pb, Cd, Co, Cu, Sb, Zn, Ti, and Ag) [36,44,45,46,47,48]. The detected levels of these contaminants were mostly below the health-based limit values for non-cancerous pathologies [17,33,49]. However, some studies warn of an additional body burden of certain (potential) carcinogens [34,36,37,42], requiring further follow-up and control to increase mask safety. One of these possible carcinogens is titanium dioxide (TiO_2_), generally present as nanoparticles (NPs), the subject of this study.

Mask manufacturers are increasingly incorporating nanofibres, nanocomposite, and nanoparticle technology into face masks, claiming improved filtration and antimicrobial and self-cleaning activity. Compared to larger particles, metal NPs often have the advantage of increased reactivity, enhanced photocatalytic activity, and improved dispersion in fibres during the production process. However, questions were raised regarding nanosafety [50,51,52]. If not firmly embedded in the fabric of the face mask, NPs may be released and inhaled. NPs’ toxicity is due to their small size (<100 nm) and large particle surface area. Some NPs produce reactive oxygen species (ROS), resulting in oxidative stress and inflammatory responses, cell damage/death, the perturbation of cell cycles, the peroxidation of lipids, DNA damage (genotoxicity), and adverse immune responses [53,54,55,56,57,58,59,60]. TiO_2_ NPs are of particular concern [58,61,62,63], as TiO_2_ is classified as possibly carcinogenic to humans by the International Agency for Research on Cancer (IARC) [64,65] (Group 2B), and as a suspected human carcinogen (Carc. 2, H351 inhalation) by the Committee for Risk Assessment (RAC) of the European Chemicals Agency (ECHA) [66]. Reviewing the toxicity of TiO_2_ NPs requires a dual approach, considering both the unique properties and behaviour of nano-sized TiO_2_ and the toxic properties of TiO_2_ independent of its size.

In this paper, the Belgian Superior Health Council’s working group reviews the usefulness and toxicity of TiO_2_ NPs in face masks. For this purpose, a review is provided of (a) the composition and filtration efficiency of different face masks; (b) the use and antimicrobial activity of TiO_2_ and other metal (nano)particles; (c) TiO_2_ nanotoxicity and health effects; and (d) data on the release of TiO_2_ NPs from masks and textiles to quantify user exposure. Based on these insights, a conservative risk assessment is performed, in compliance with the precautionary principle.

## 2. Methodology: Literature Review and Risk Assessment

In 2023, a multidisciplinary working group was set up by the Belgian Superior Health Council. A deontological committee evaluated the risks of conflicts of interest for each participant. Given the broad and multifaceted nature of the research question, a narrative review approach was performed. Narrative reviews are flexible and practical for topics that require a synthesis of complex and broad evidence and need a detailed, nuanced description and interpretation [67]. Peer-reviewed publications and reports of national health institutions were retrieved for each sub-aspect, using databases such as PubMed, Web of Science, and the search engine Google Scholar, supplemented by relevant references from these publications. The search strategy and selection criteria varied depending on data availability and subtopic importance. For example, studies published after the start of the COVID-19 pandemic were considered for the composition of face masks and their effectiveness, while also older studies were deemed for the toxicological aspects of TiO_2_ NPs. Meta-analyses, systematic reviews, and narrative reviews were preferred, while experimental studies were used to illustrate specific aspects, nuances, or insights. Depending on their availability, the same rationale was followed for the inclusion of experimental studies on the use and release of TiO_2_ NPs.

Based on the available literature, a conservative risk assessment is provided, following the precautionary principle. First, the potential TiO_2_ exposure was calculated based on the highest measurements in two published leaching experiments. A theoretical Time-Weighted Average (TWA) of TiO_2_ NP-inhalation exposure was derived for four long-term, worst-case exposure scenarios with intensive mask use. Finally, the Risk Characterisation Ratio (RCR) was calculated using several international (sub)chronic exposure limits.

## 3. Mask Types, Composition, and Filtration Efficiency

**Figure 1:**
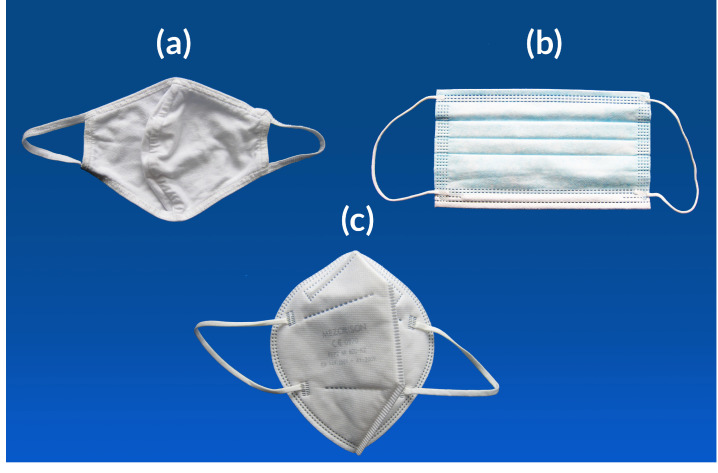
Different mask types: (**a**) cloth mask, (**b**) surgical mask, and (**c**) respirator.

Before investigating the added value of “biocidal” metal (nano)particles, the composition of face masks and their efficiency as physical barriers that retain infectious droplets need to be reviewed. Various types of masks can be distinguished, each with different target users, filtration efficiency, and legal requirements. While definitions of “face masks” often exclude filtering facepiece respirators, they are included here, following several reviews and meta-analyses on their use during the COVID-19 pandemic [6,7,8,9,10,11,12,13,14,15,16]. Three main types can be distinguished (Figure 1).

- *Cloth masks*: Reusable homemade face masks for non-medical use, made of cotton [9]. Sometimes, other fabrics are used, such as silk, flannel, synthetics, and combinations of these [68]. Due to this variation, the performance of cloth masks is very heterogeneous: filtration efficiencies for a single layer of various fabrics ranged from 5 to 80% for particle sizes < 300 nm, and from 5 to 95% for particles > 300 nm [68]. Mechanical filtration can be enhanced by combining multiple layers and using cotton with high weave densities (Table 1) [68]. During the earliest part of the COVID-19 pandemic, shortages of surgical and N95 masks occurred, leading local governments to call on citizens to manufacture cloth masks [9,11,69,70]. Due to the lack of control and standardisation, the safety of these masks raises questions.
- *Surgical masks*: Disposable, professionally produced face masks consisting of three or four nonwoven layers, mainly intended for medical use by infected patients [71,72]. During the COVID-19 pandemic, surgical masks became widely used by the general public. Both surgical masks and respirators are composed of a variety of thermoplastic materials (e.g., polypropylene, polyurethane, polyacrylonitrile, polystyrene, polycarbonate, polyethylene, and polyester) [47,73]. Three-ply surgical masks consist of a hydrophobic external layer repelling mucosalivary droplets, a filtering middle layer (usually polypropylene), and a skin-friendly inner layer that retains droplets from the user [9,71]. In four-ply masks, an additional filtering layer is added, sometimes with activated carbon [71]. In general, the high-density fibre construct used for the outer and inner layers is produced via melt-spinning, while the filtering middle layer requires finer microfibres produced through melt-blowing [9]. Surgical masks perform better than cloth masks in terms of filtering capacity (Table 1). They are certified according to the American ASTM F2100 and European EN 14 683:2019 standards [11]. European types I and II have a Bacterial Filtration Efficiency (BFE, determined via *Staphylococcus aureus* aerosol) of >95 and 98%, respectively, while IR and IIR masks are also splash-resistant. Similarly high Viral Filtration Efficiencies (VFE > 98%) were obtained by Whiley et al. [74]. In the latter study, *S. aureus* (~1 µm) was replaced by bacteriophage MS2 (~27 nm), which is 2–3 times smaller than the SARS-CoV-2 virus [74].
- *Respirators*. Both reusable and disposable, professionally produced, highly performant protective devices to prevent the inhalation of dust particles, aerosols, and infectious agents. Filtering facepiece (FFP) respirators are intended to protect healthcare workers during contact with patients with airborne diseases, such as COVID-19 or influenza [71,72]. Unlike surgical and cloth masks, respirators are fitted tightly against the face, forcing particles through the filtering material. Filtration is mainly achieved mechanically, due to the polypropylene microfibres, and through electrostatic attraction [71]. In the European Union, three types of disposable Filtering Facepiece respirators exist (FFP1, FFP2, and FFP3), certified under the European Standard EN 149:2001 + A1:2009. They have minimum filtration efficiencies (at 95 L/min air flow) of at least 80%, 94%, and 99%, and a maximum inward leakage of less than 22%, 8%, and 2%, respectively. In the US, the National Institute for Occupational Safety and Health (NIOSH) approves N95 respirators that achieve a minimum of 95% filtration efficiency at approximately 300 nm NaCl aerosol size, certified under the NIOSH 42 CFR 84 standard [75,76]. Chinese KN95 respirators also match similar criteria under the GB2626 standard, filtering at least 95% of particles around 300 nm. Hence, N95, K95, and FPP2 respirators are very similar [11,71]. Despite some product-specific exceptions [77], measurements confirm the very high filtration requirements, often performing > 99% for particles > 300 nm (Table 1). Zhou et al. [78] demonstrated a > 99.7% efficiency of a new N95 mask for the exclusion of the influenza A virus, rhinovirus 14, and *S. aureus*. While the filtration requirements of FFP2, N95, and K95 respirators are fixed, their structure and composition may vary by brand [11,70,78].

A good understanding of the behaviour of large respiratory droplets and airborne transmission is important to optimise preventive measures against respiratory viruses. While large particles (>20 µm) fall on the ground due to gravity, particles below 5–10 µm are prone to be inhaled, while aerosol particles < 1 µm may stay in the air for 12 h [10,70,79,80]. The main mechanisms for the filtration of aerosols are gravity sedimentation, inertial impaction, interception, diffusion, and electrostatic attraction. The first two are most important for particles between 1 and 10 µm [68,81].

As the individual size range of SARS-CoV-2 varies from 0.07 to 0.09 µm, the minimum size of a respiratory particle to contain the virus was initially calculated to be ca. 9.3 µm [82]. However, more than 90% of the viral RNA exhaled during vocalisation was found in aerosol particles < 4.5 µm, with the highest concentrations between 0.94 and 2.8 µm [83]. This is due to evaporation, as the diameter of the original droplets is assumed to be up to five times larger [83]. Hence, surgical masks and FFP2/N95/KN95 respirators provide effective protection, especially against large droplets (Table 1). Infected persons should wear surgical masks to protect people in their immediate vicinity, combined with continuous ventilation of their rooms to remove the finest aerosols, which are not retained. The more “gaps” between the surgical mask and the user’s face, the more the effectiveness decreases. In turn, healthcare workers should protect themselves by using tight-fitting respirators as personal protective equipment (PPE). While the evidence is still uncertain [84,85], Chu et al. [8] found that N95 respirators are indeed more protective against COVID-19/SARS/MERS infection (aOR 0.04, 95% CI 0.004–0.30) than surgical masks (aOR 0.33, 95% CI 0.17–0.61). Calculating one-to-one exposure, Bagheri et al. [15] drew similar conclusions: even loosely fitted FFP2 respirators may reduce the infection risk by a factor of 2.5 compared to tight-fitting surgical masks. These authors also concluded that when both persons wear a mask (surgical and/or FFP2), the transmission of COVID-19 is effectively minimised [15]. While cloth masks are not sufficient for professional use, they seem suitable to reduce viral circulation among the general public if they contain multiple cotton layers of high weave density [68] (Table 1).

**Table 1:** Experimental filtration efficiencies of different mask types reported in the literature [68,74,86,87]. Remarks: 1 CFM (cubic feet per minute) = 1.699 m^3^/h. TPI = threads per inch. VFE = Viral Filtration Efficiency.

Mask Type	Study	Particle Size (nm)	Filtration Efficiency (%)	Remarks
Cloth masks	Rengasamy et al. [87]	20–1000, median 75 ± 20	10–26	Polydisperse NaCl aerosol. Face velocity: 5.5 cm/s.
	Konda et al. [68]	<300	9 ± 13	1 layer quilter’s cotton (80 TPI). Polydisperse NaCl aerosol, 1.2 CFM.
			38 ± 11	2 layers quilter’s cotton (80 TPI). Polydisperse NaCl aerosol, 1.2 CFM.
			79 ± 23	1 layer cotton (600 TPI). Polydisperse NaCl aerosol, 1.2 CFM.
			82 ± 19	2 layers cotton (600 TPI). Polydisperse NaCl aerosol, 1.2 CFM.
		>300	14 ± 1	1 layer quilter’s cotton (80 TPI). Polydisperse NaCl aerosol, 1.2 CFM.
			49 ± 3	2 layers quilter’s cotton (80 TPI). Polydisperse NaCl aerosol, 1.2 CFM.
			98.4 ± 0.2	1 layer cotton (600 TPI). Polydisperse NaCl aerosol, 1.2 CFM.
			99.5 ± 0.1	2 layers cotton (600 TPI). Polydisperse NaCl aerosol, 1.2 CFM.
	Sankhyan et al. [86]	300	16–23	Ammonium sulphate aerosol. NIOSH N95 filtration efficiency procedure.
Surgical masks	Konda et al. [68]	<300	76 ± 22	No gap. Polydisperse NaCl aerosol, 1.2 CFM.
			50 ± 7	With gap. Polydisperse NaCl aerosol, 1.2 CFM
		>300	99.6 ± 0.1	No gap. Polydisperse NaCl aerosol, 1.2 CFM.
			44 ± 3	With gap. Polydisperse NaCl aerosol, 1.2 CFM
	Sankhyan et al. [86]	300	42–88	Ammonium sulphate aerosol. NIOSH N95 filtration efficiency procedure.
	Whiley et al. [74]	Average: 2600	98.5, 99.5	Average VFE _(2.6 µm)_ calculated with larger aerosols excluded. Adapted ASTM F201-14 method with MS2 bacteriophage.
		Average: 6000	99.6, 99.9	Average VFE _(6.0 µm)_. Adapted ASTM F201-14 method with MS2 bacteriophage.
Respirators (N95, K95)	Rengasamy et al. [87]	20–1000, median: 75 ± 20	99.88	Polydisperse NaCl aerosol. Face velocity: 5.5 cm/s.
		20–1000, median: 75 ± 20	>95	Polydisperse NaCl aerosol. Face velocity: 16.5 cm/s.
	Konda et al. [68]	<300	85 ± 15	No gap. Polydisperse NaCl aerosol, 1.2 CFM.
		>300	99.9 ± 0.1	No gap. Polydisperse NaCl aerosol, 1.2 CFM.
	Sankhyan et al. [86]	300	83–99	Ammonium sulphate aerosol. NIOSH N95 filtration efficiency procedure.
	Whiley et al. [74]	Average: 2600	99.3	Average VFE _(2.6 µm)_ calculated with larger aerosols excluded. Adapted ASTM F201-14 method with MS2 bacteriophage.
		Average: 6000	99.9	Average VFE _(6.0 µm)_. Adapted ASTM F201-14 method with MS2 bacteriophage.

## 4. Metal (Nano)Particles in Face Masks: Application and Antimicrobial Properties

Metals and metalloids have been found and quantified in both surgical masks and respirators in a multitude of studies [44,46,47,88,89,90,91]. Some are contaminants from the production process, as different steps of the polymer processing require heavy metal and metalloid catalysts (e.g., Sb oxides/acetates; Ti and Zr compounds; Sn complexes), additives for flame-retardants (e.g., Sb and Al oxides), pigments (e.g., Pb, Cd, Cr, Cu compounds), or stabilisers (e.g., Pb, Cd compounds) [47]. Metals are also intentionally incorporated into the polymer fabric and attached within and/or on the fibres to fabricate masks with antimicrobial properties or different quality/cosmetic aspects (UV protection, whitening, etc.) [6,9,36,46,92,93]. Silver ions (Ag^+^), large silver particles (Ag^0^), silver nanoparticles (Ag NPs), copper oxide nanoparticles (CuO NPs), zinc oxide nanoparticles (ZnO NPs), titanium dioxide nanoparticles (TiO_2_ NPs), and combinations of these are commonly used for antimicrobial purposes [6,44,46,70,88,89,92,93]. In practice, some particles may exceed the defined limit of 100 nm [46], which means that, strictly speaking, nano- and small microparticles (MPs) can be found together. Metals can be incorporated into the fibres of the fabric as a nanocomposite but are also often present as surface coatings [88,89,93].

While each compound has a slightly different mode of action and effectiveness [9,93,94], two general biocidal mechanisms are distinguished [9]: (1) metal ions can damage key functions in the cell wall or viral envelope by binding and precipitating thiol groups in proteins, phosphate groups in ATP or DNA, and other groups with a negative charge; and (2) the generation of ROS and induction of oxidative stress due to changes in the redox states and photocatalytic activity [9]. NPs also complicate viral attachment to the host cell [95]. Recently, a nano-quantitative structure–activity relationship (QSAR) model was proposed to predict the cytotoxicity of different metal oxide NPs in *Escherichia coli*, allowing for a quantitative comparison between them [94]. In the following paragraphs, the most important antimicrobial metals used in face masks are reviewed. A general summary is provided in Table 2.

### 4.1. Silver

Silver is well-known for its antimicrobial effects against bacteria, viruses, and fungi [6,48,95]. It has a broad-spectrum biocidal activity through contact, strongly influenced by its physicochemical properties, and is widely applied in coatings of medical equipment [9,95]. In face masks, Montalvo et al. [89] and Mast et al. [44] distinguished four types of silver-based biocides: (1) Ag^+^ ions, (2) Ag NPs within the fibre matrix, (3) Ag NPs and large Ag particles at the surface of, or close to, cotton fibres in masks containing polycationic polymers binding Ag^+^ ions, and (4) coatings of metallic silver releasing Ag^+^ ions, Ag NPs, and large silver particles [44,89]. In this study, the total amount of silver ranged between 3 and 235 µg/mask. Only 4 of the 13 tested masks with silver contained Ag NPs, of which one was silver-coated [44].

Ag NPs have a multifaceted mode of action, penetrating and damaging cells. The main advantage of Ag NPs is their ability to continuously release silver ions via dissolution, while the biocidal action of the latter is not light-dependent [95]. Excessive ROS generation and silver–thiol (R-SH) actions are the main drivers for the antimicrobial effects of nano-silver, which damages the cell membrane, proteins, lipids, and DNA [93]. The virucidal mode action is similar and primarily driven by ROS production, leading to viral replication inhibition, direct viral inactivation, binding of the virus, and DNA interactions [96]. Along with other proteins, Ag NPs can inhibit glycine and alanine of SARS-CoV-2’s S-protein, an ideal target for antiviral action [97]. In *E. coli*, the nano-QSAR-predicted log (1/EC_50_) value of Ag_2_O NPs is 4.07, indicating high cytotoxicity [94].

An experiment with metal NPs embedded in polyacrylonitrile nanofibres showed that silver has a high level of antibacterial activity, while ZnO and TiO_2_ displayed no bactericidal effects [98]. Botelho et al. [99] described the effectiveness of a nylon fabric coated with Ag NPs (average 25 nm) and chitosan (acting as a surfactant for the NPs) in reducing *S. aureus* and *Pseudomonas aeruginosa*. The coating was found to be successful for single-use face masks. In addition, 4 nm Ag NPs deposited onto a face mask can neutralise up to 98% of virions reaching the mask [100].

### 4.2. Copper

Copper is a highly effective contact killer of microorganisms, used in disinfection, crop protection, construction, medicine, water/liquid treatment, and textile industries [9,93,96]. Also, CuO NPs can release copper ions. Copper-derived NPs are cheaper and more stable than Ag NPs [93]. They can be integrated in the textile via microencapsulation, allowing for a slower release than when they are applied as coatings [93]. Pollard et al. [52] detected between 2 and 14 mg Cu in five masks. Combinations with other metals are also observed: one mask is described to be treated with a preservative containing 93.337% CuO (much in NP form), 0.313% Zn, and 0.007% Ag [44,89].

Early in the COVID-19 pandemic, Van Doremalen et al. [101] described the high effectiveness of Cu against SARS-CoV-2, as no viable virus was measured after 4 h of contact on a copper surface. Copper damages the plasma membrane as it is redox-active and induces the formation of free radicals that promote lipid peroxidation [93,102]. Moreover, proteins and genetic material are altered. The nano-QSAR-predicted log (1/EC_50_) value of CuO NPs in *E. coli* is 3.35, indicating lower bacterial cytotoxicity compared to silver [94]. Copper NPs disrupt viral integrity and cross-link and bind with DNA strands. In this way, the viral genome is destroyed [95].

The effectiveness of CuO NPs in a respirator against influenza viruses is illustrated by Borkow et al. [103]: within 30 min, the NPs almost completely reduced infectious influenza A virus titers on the mask surface. In addition, Giedraitienè et al. [104] demonstrated a bactericidal effect of CuO NPs on a medical mask for both Gram-positive and Gram-negative bacteria.

### 4.3. Zinc

Nanomaterials of ZnO have diverse applications in biomedicine, energy storage, electronics, optics, etc. [96,105,106]. ZnO NPs show effective, size-dependent antimicrobial activity [107]. ROS generation by Zn NPs and the release of Zn^2+^ ions cause lipid peroxidation and membrane damage, while proteins are inactivated and DNA damaged within the bacterial cell [93,96,106,108,109]. Zinc shows antiviral activity against a broad spectrum of viruses (SARS-CoV-19, HIV, HPV, HCV, HEV, RSV, HSV, and EAV), effectively inhibiting viral replication [95]. Concerning cytotoxicity in *E. coli*, the QSAR-predicted log (1/EC_50_) value of ZnO NPs is 3.39, lower than in silver NPs, similar to CuO NPs [94]. Unlike silver and copper NPs, which exert their effects mainly by releasing ions, the antimicrobial action of zinc NPs is partly caused by its photocatalytic activity, producing ROS via light-catalysed redox reactions [9,48,92]. Compared to TiO_2_, ZnO proved to be a faster and more effective photocatalyst for the inactivation of *E. coli* in water [110]. As ZnO has a band gap energy of 3.37 eV at 300 K, ZnO nanostructures need UV light for optimal photocatalytic performance [111].

Indeed, without continuous UV light, nanofibres with ZnO showed no significant bactericidal activity [98]. To achieve better antimicrobial results, nano-zinc is often combined with other metals, including silver [93]. Recently, the effective use of a novel zinc–ion-embedded fibre in a nonwoven disposable mask was described [112]. Cu_2_O-doped-ZnO NPs enclosed in a polydopamine shell showed a good antibacterial effect in surgical masks, even at a low metal loading [113].

### 4.4. Titanium Dioxide

TiO_2_ is used in many industrial and cosmetic applications, including sunscreen and textiles, due to its excellent UV-absorbing properties and chemical and biological stability [46,48,114,115,116,117,118,119]. It improves the stability of textiles against UV light and acts as a whitening colourant and matting agent, making fibres opaque in polyester and polyamide fabrics [46,115]. It is one of the most frequently applied photocatalysts, achieving effective ROS-induced antimicrobial action with the appropriate radiation [95,116]. In a recent study, near-spherical TiO_2_ (nano)particles (median sizes 89–184 nm) were detected in at least one layer of single-use and reusable face masks, incorporated in different synthetic fibres [46]. In this study, the total mass of TiO_2_ strongly varied in each mask, ranging from 791 to 152,345 µg with 17 to 4394 µg at the fibre surface [46]. In another study, traces of Ti were found in all studied FFP2 and surgical masks [90].

**Figure 2:**
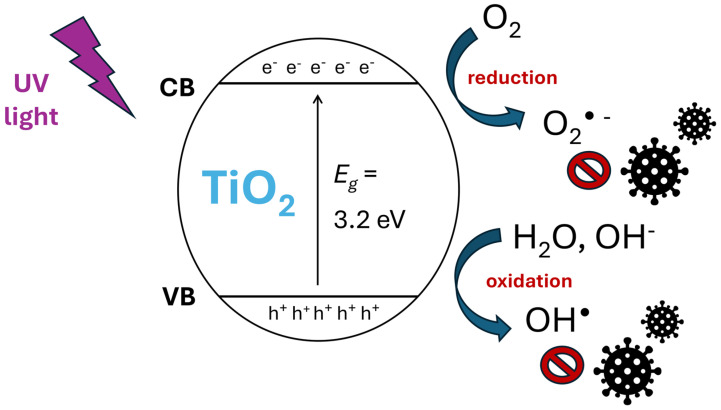
Antimicrobial effect via ROS generation through photocatalysis at the surface of TiO_2_ NPs. CB = Conduction Band. VB = Valence Band [93,96,117,118,119,121,122,123,124].

While the antimicrobial mode of action of other metal NPs largely occurs through the release of ions, this is not the case for TiO_2_ NPs [120]. The dominant antimicrobial mechanism is ROS generation through photocatalysis. When TiO_2_ (anatase) absorbs photon energy equal to or higher than its band gap (≥3.2 eV), electrons from the valence band (VB) are excited to the conduction band (CB), forming electron–hole pairs (e^−^ _CB_/h^+^ _VB_). The excited electrons in the CB (e^−^ _CB_) reduce adsorbed O_2_ to superoxide radicals (O_2_^•−^), while the holes in the VB (h^+^ _VB_) are strong oxidizers that can react with H_2_O or hydroxide ions (OH^−^) on the TiO_2_ surface to form hydroxyl radicals (OH^•^) [93,96,117,118,119,121,122,123,124] (Figure 2). After attachment to the TiO_2_ NPs via electrostatic force, the generated ROS damage bacteria and viruses externally (cell membranes and viral envelopes) and internally (DNA oxidation, protein denaturation, mitochondrial damage, etc.) [50,93,125]. Compared to other metal NPs, the QSAR-predicted log (1/EC50) of TiO_2_ NPs (1.95) indicates relatively low cytotoxicity in *E. coli* [94].

In general, TiO_2_ is most abundant in two tetragonal forms (anatase and rutile) [116,118,126]; both have wide energy band gaps (*E_g_* = 3.20 and 3.02 eV) requiring UV light for photocatalytic activity [116,117]. However, only ca. 3% of the sunlight at the Earth’s surface can be used by TiO_2_, resulting in low activity, which even decreases in (artificial) indoor light [116,117,127]. For this reason, several strategies are researched and implemented to correlate the photoresponse of TiO_2_ with the visible solar spectrum, including metal (e.g., silver) and non-metal (e.g., carbon and nitrogen) doping, or surface modification with noble metal NPs [116,117,118,119,127,128,129]. For instance, sunlight-irradiated Mn-doped TiO_2_ NPs could reduce the presence of *Staphylococcus aureus* and *Klebsiella pneumoniae* in cotton within 120 min by 100% [130].

Multiple studies discuss the use of TiO_2_ NPs for antimicrobial purposes in face masks or textiles; some examples are summarised by Bhandari et al. [93]. Without continuous UV irradiation, no significant bactericidal activity was observed in polyacrylonitrile nanofibres with TiO_2_ [98]. In contrast, Ahmed and Alamro [131] reported strong antibacterial activity for a face mask coated with high concentrations of TiO_2_ NPs (2% solution). To improve biocidal effectiveness, TiO_2_ is often combined with other metals, such as silver [46,88,89,123,132,133]. Given the intrinsic toxicity of NPs, Margarucci et al. [125] examined whether micrometric TiO_2_ particles could be a safer alternative in face masks. Surprisingly, the microparticles (MPs) outperformed the NPs in reducing *E. coli*. The use of TiO_2_ MPs under blue light was concluded to be a promising alternative [125].

## 5. Metal (Nano)Particles in Face Masks: Legal Status in the European Union

When metal (nano)particles are used for antimicrobial purposes in the European Union, they must comply with Regulation (EU) No 528/2012 on the marketing and use of biocidal products. According to Article 19 of the Regulation, the risks to human, animal, and environmental health need to be assessed separately if nanomaterials are used in a product considered for authorisation. Annex V of Regulation (EU) No 528/2012 distinguishes 22 different product types (PTs), of which three categories are relevant for face masks: PT01 (“*human hygiene*”), PT02 (“*disinfectants and algaecides not intended for direct applications to humans and animals*”, including “*products used to be incorporated in textiles, tissues, masks, paints and other articles or materials with the purpose of producing treated articles with disinfecting properties*”), and PT09 (“*Fibre, leather, rubber and polymerised materials preservatives*”, including “*products used for the preservation of fibrous or polymerised materials, such as leather, rubber or paper or textile products by the control of microbiological deterioration*”).

Based on the ECHA Biocidal Active Substances Database (accessed on 23 January 2025), silver zinc zeolite is allowed (PT02, -09), while many applications of copper and silver (PT01, -02, and -09) are no longer supported, not approved, or under evaluation. In 2021, the Commission Implementing Decision (EU) 2021/1283 banned Ag NPs in PT02 and PT09 applications. Hence, the use of Ag NPs for biocidal purposes has lost its legal basis regarding their use in face masks. A “*reaction mass of titanium dioxide and silver chloride*” and the applications of “*silver chloride deposited on titanium dioxide*” are no longer supported or under evaluation by the competent authorities. Presumably, only the silver is intended as a biocide, while TiO_2_ is included for UV stability or formulation purposes. To conclude, many “antimicrobial” face masks rely on specific applications of metal (nano)particles (Ag, Cu, Zn, TiO_2_) that have not been submitted for approval, are no longer authorised, or have not (yet) been authorised for biocidal purposes in masks/textiles in the EU.

## 6. Hazard Identification and Characterisation of TiO_2_ Nanoparticles

The hazard characterisation of TiO_2_ (regardless of particle size) and its regulatory scrutiny have evolved considerably in recent decades. Due to its stability, poor bioavailability, and few observed adverse effects in relevant concentrations among humans and nontarget organisms, the US Environmental Protection Agency (EPA) classifies pigment grade TiO_2_ as a List 4B inert ingredient [134]. However, increasing evidence shows that this does not apply to nanoscale TiO_2_ [58,61,62,63,119,126,135,136,137,138,139,140,141].

The toxicity of NPs depends on both their surface chemistry and nanosized formulation. Particle size, shape, surface area, surface charge, surface binding locations for organic molecules, and crystallinity are deterministic for NP toxicity [57,59,60,63,137,142,143,144]. Consequently, these characteristics should be incorporated into the toxicological evaluation of TiO_2_ NP applications. The synthesis method (e.g., sol-gel, hydrothermal, solvothermal, and multiple other techniques) and associated parameters (e.g., temperature and pressure) largely determine these properties [145]. Furthermore, the increasing use of metal- and non-metal-doped TiO_2_ NPs, along with various surface modifications [116,117,118,119,127,128,129], introduces additional challenges for future toxicological assessments.

**Figure 3:**
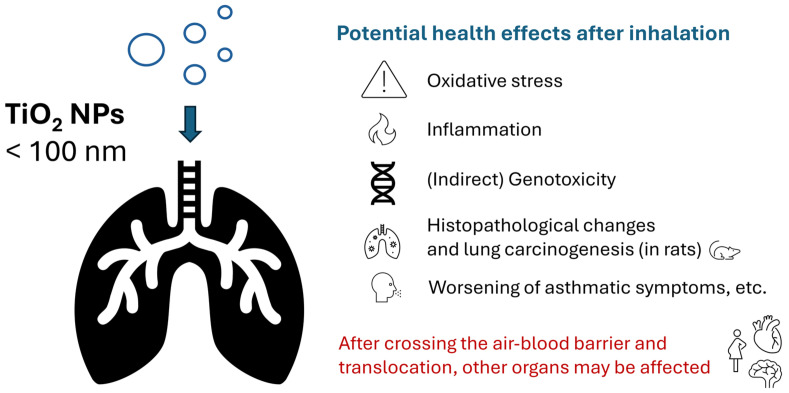
Potential health effects after the inhalation of TiO_2_ NPs. A more detailed overview is provided in the text.

In the following sections, key insights are summarised concerning the toxicity and health effects of TiO_2_ NPs, mainly after inhalation (Figure 3). This is not intended to provide completeness, as many uncertainties remain.

### 6.1. Oxidative Stress

While its toxicokinetics in the human body are still poorly understood, TiO_2_ NPs mainly enter the cell via active endocytosis (phagocytosis and pinocytosis) and passive diffusion [62,63,136]. In lung cells, TiO_2_ NPs were detected in the cytosol, especially in the peri-region of the nucleus, in vacuoles, lamellar bodies, and lysosomes [137]. Oxidative stress is the key mechanism of nanotoxicity, acting through excessive ROS production, occurring both with and without the photo-activation of TiO_2_ NPs [58,62,63,135,136] (Figure 2). When the generated radicals exceed the capacity of cellular antioxidant defences, cells are damaged. For example, lipid peroxidation, oxidative DNA damage, micronuclei formation, increased nitric oxide, and hydrogen peroxide production can occur in human bronchial epithelial cells [146]. In addition, ROS formation affects cellular signalling for cell proliferation, inflammation, and cell death [63,138].

### 6.2. Genotoxicity

Gene mutation, chromosomal damage, and aneugenicity are assessed through many in vitro and in vivo tests and mathematical modelling. As each test does not cover all endpoints, the outcomes can be contradictory and difficult to interpret [137,140,147,148]. Concerning the genotoxic effects of TiO_2_ as a food additive (E171, both NPs and MPs), a cut-off value for the particle size could not be identified by the European Food Safety Authority (EFSA) [149]. TiO_2_ NPs have the potential to induce DNA strand breaks and chromosomal damage, but nearly all mutagenicity tests are negative [148,149]. Even short-term exposure to TiO_2_ NPs can cause genotoxicity in vitro [140]. As different modes of action may operate in parallel and these are still poorly understood [150], it is still uncertain if a threshold mode of action can be assumed [149]. In vitro and in vivo studies indicate that the genotoxic effects of TiO_2_ NPs are mainly due to secondary mechanisms such as oxidative stress, related to their small particle size and large surface area [63,66,141,148,150,151]. However, direct DNA interactions and subsequent genetic damage have also been described, and require further study [140,150,152].

### 6.3. Respiratory Toxicity (Non-Carcinogenic)

During inhalation, TiO_2_ NPs and MPs are distributed throughout the respiratory tract. Large particles (0.5–10 µm) remain on the epithelium of the airways and the alveoli [135,153]. Half of the 20 nm particles are distributed in the alveolar region. The fraction between 1 and 5 nm is distributed throughout the nasopharyngeal, tracheobronchial, and alveolar regions. Of the most fine particles of 1 nm, 90% end up in the nasopharyngeal region, and 10% end up in the tracheobronchial region [135,153]. Another study measured both total and regional lung deposition for different sizes of ultrafine particles (no TiO_2_) [154]. The peak deposition of NPs occurred in the transition zone between the conducting airways and the alveolar region, while proximal airway regions received the largest surface dose, which amounts to a value several times greater than the average lung dose [154].

Given the large surface area (40–100 m²) of the ca. 300 million alveoli and their thin barrier (ca. 0.5 µm) with the capillaries [63,155], human alveoli are sensitive to toxic exposure. After a portion of the inhaled TiO_2_ NPs reaches the alveolar region, some NPs may cross the air–blood barrier, enter the bloodstream, and be transported to other organs [63]. The translocation of TiO_2_ NPs from the pulmonary airways into other pulmonary compartments or systemic circulation is still debated and requires further research [58].

While insoluble particles are predominantly cleared in the upper respiratory tract via the mucociliary escalator, the main alveolar clearance mechanism is macrophage phagocytosis [156]. Alveolar macrophages of rats were shown to clear TiO_2_ MPs (ca. 3–6 µm), but difficulties arise with NPs (ca. 20 nm). Moreover, the clearance of NPs is slower than that of larger ones (200 nm) [63]. In rats, long-term exposure to relatively high concentrations of TiO_2_ (both NPs and MPs) can result in impaired clearance, leading to lung overload. This leads to the continuous production of neutrophils, the activation of cytokine production, and persistent inflammation of macrophages and epithelial cells [63,135,157]. Compared to impaired clearance, inflammation is also induced in rats at lower cumulative doses via ROS generation and oxidative stress [157]. Inflammatory responses after acute exposure seem to be modest and reversible in multiple studies, regardless of particle size [58,63,136].

In Sprague Dawley rats, repeated exposure to TiO_2_ NPs via intra-tracheal instillation has led to different histopathological changes: 0.5 mg/kg bw resulted in slight lymphocyte and macrophage aggregation, pulmonary emphysema, macrophages accumulation, and alveolar septa disruption [158]. At 4 mg/kg bw, slight inflammation was observed, along with lymphocyte and macrophage aggregation, alveolar wall thickening, terminal bronchiole collapse, and interstitial thickening [158]. Similar observations were reported in mice [159], and fibrosis has also been described [58,62].

The doses administered in these rodent studies are generally much higher than those representative of human exposure. The relevance of the observed sensitivity in rats for human risk assessment remains debated. According to Braakhuis et al. [157], humans are less sensitive than rats for multiple reasons, as follows: (1) the clearance capacity of human lungs is estimated to be seven times higher than that of rat lungs, based on the number and volume of alveolar macrophages and the volume of the lung lining fluid; (2) in humans, more particles are deposited in the interstitium, and interstitial macrophages are less inflammogenic than alveolar macrophages; (3) human alveolar macrophages lack nitric oxide synthetase, reducing their inflammatory responses compared to rats [157]. The lower sensitivity of humans to poorly soluble low-toxicity particle (PSLT)-induced lung inflammation was also highlighted by the ECHA RAC assessment [66]. However, Skocaj et al. [63] estimated that the doses leading to lung overload in rats might be relevant for highly exposed workers, based on (1) indications that the lung clearance of poorly soluble particles may be slower in humans than in rats and mice [160], while (2) the response of lung tumours to nonsoluble particles can be predicted based on particle surface area, without accounting for overloading [141].

Several studies show additional sensitivity to TiO_2_ NPs in asthmatics, affecting the severity of symptoms [58,138]. Furthermore, exposure in the early stages of lung development might increase the risk of developing asthma, highlighting the importance of protecting infants [58].

Differences in respiratory toxicity have been observed between the different polymorphid forms of TiO_2_, with anatase turning out to be more toxic than rutile, as well as in human lung epithelial cells [126,137,161,162,163]. Inflammatory effects after both acute and chronic exposure are more pronounced for smaller particles. The dose–response relations in nanotoxicology do not primarily depend on mass dose, but rather on other dose metrics such as particle surface area, although some studies failed to observe this relationship [58,135].

### 6.4. Lung Carcinogenesis

The most controversial endpoint is lung carcinogenicity. In 2006, IARC classified TiO_2_ (regardless of size) as “possibly carcinogenic to humans” (Group 2B) [64,65]. Sufficient evidence for the development of lung tumours was found by IARC in inhalation studies with rats [164,165,166,167], and experiments with intratracheally exposed rats [168]. A higher incidence of both benign and malignant lung tumours was observed, especially in highly exposed groups. The average nanoparticle concentration in the study of Heinrich et al. [166] was 10 mg/m^3^ (P25 Degussa TiO_2_ NPs). Interestingly, no such effects were observed among mice and hamsters. In addition, the epidemiological evidence was evaluated to be inadequate for carcinogenicity in humans [64]. Few qualitative epidemiological studies exist, with only a moderate confidence level [169]. Furthermore, TiO_2_ exposure in these epidemiological studies is usually not limited to nanosized particles. While one multicountry cohort study of predominantly pigment-grade TiO_2_ production workers showed a slightly increased risk for lung cancer (SMR 1.23, 95% CI 1.10–1.38) (but no dose–response relation) [170], other cohort studies [171,172] and community-based case–control studies [173,174] did not find a statistically significant increase in lung cancer odds ratio (OR) or standardised mortality ratio (SMR). Later, other Canadian case–control [175] and US cohort studies [176,177] failed to detect an excess risk of lung cancer mortality. A nonsignificant summary SMR of 1.10 (95% CI 0.91–1.32) was found for lung cancer in the meta-analysis of Le HQ et al. [178].

NIOSH and ECHA drew similar conclusions to IARC. NIOSH determined that ultrafine TiO_2_ (NPs, <100 nm) is a potential occupational carcinogen, but the evidence was insufficient to conclude the same for fine TiO_2_ (MPs, >100 nm), as epidemiological studies often lack the statistical power to detect weak carcinogens [141]. When the administered doses of fine and ultrafine particles (MPs and NPs) are expressed as total particle surface area in the lungs, NIOSH concluded that both fit on the same dose–response curve for rat tumours [141]. In Europe, the RAC of ECHA classified TiO_2_ as a substance suspected of causing cancer through the inhalation route (Carc. 2, H351 inhalation) [66]. In their comprehensive opinion, human and animal studies were weighted and uncertainties were considered (e.g., lung overload and interspecies differences) [66].

Although direct DNA damage cannot be ruled out, it is generally accepted that the carcinogenic activity of TiO_2_ NPs is mainly indirect, due to secondary genotoxicity related to the particle size and their large surface area [66,141,151,179,180,181]. The exact carcinogenic mechanism remains to be further elucidated. Moderate to high evidence exists regarding genotoxicity, oxidative stress, and chronic inflammation, while the evidence remains inadequate for epigenetic changes, receptor-mediated effects, altered proliferation, and cell death [182]. Over time, our understanding can be improved through the inclusion of new evidence in the Adverse Outcome Pathways (AOPs) [183]. These provide the sequence of molecular and cellular events from exposure to the development of neoplastic lesions. Braakhuis et al. [157] proposed an AOP for the prolonged inhalation of TiO_2_ (both NPs and MPs), mainly based on rat studies. Impaired clearance was identified as the initiating event, followed by seven key events. ROS generation, oxidative stress, and persistent inflammation are at the base of the sequence, leading to epithelial injury, regenerative cell proliferation, and hyperplasia. Ultimately, these processes may result in tumour development [157]. Until the relevance of impaired clearance in humans is elucidated, Bos et al. [184] concluded that observations in rats should be considered relevant for human risk assessment, following the precautionary principle.

### 6.5. Other Health Effects

Many other effects have been related to different routes of TiO_2_ NP exposure, as they are capable of damaging a variety of cell types [137] and can be transported to different organs [58]. Immunotoxic effects are possible due to the uptake of NPs by macrophages, monocytes, platelets, leukocytes, and dendritic cells, triggering inflammatory responses [58,63]. TiO_2_ NPs can also translocate to the central nervous system through the olfactory pathway, crossing the blood–brain barrier and causing pathological changes that can potentially lead to neurotoxic effects [62,63,136]. Worryingly, TiO_2_ NPs can be transported from the mother to the foetal brain, with possible effects on its development, highlighting an additional risk in the early life stages and pregnancy [58]. Cardiovascular effects are also known to occur, triggered by oxidative stress and inflammation [136]. TiO_2_ NPs can disturb mitochondrial functioning, accelerate atherosclerosis, and disturb the cardiac autonomic function [63,136]. Hepatotoxicity was demonstrated in multiple in vivo studies, indicated by several serum biochemical parameters [58,185]. Also, endocrine disruption has been shown in different animal studies, including altered hormone levels in mice [186]. TiO_2_ NPs have the potential to accumulate in reproductive organs, damaging the development of the ovum and sperm, while potentially affecting the offspring after crossing the blood–testis and placental barriers [187]. The main mechanisms for reproductive toxicity are described to be oxidative stress, irregular cell apoptosis, inflammation, genotoxicity, and hormone synthesis disorder [187]. However, it remains unclear if humans are at risk under realistic exposure scenarios [135].

### 6.6. Health-Based Inhalation Exposure Limits for TiO_2_ NPs

In 1993, the US Occupational Safety and Health Administration (OSHA) established a high Permissible Exposure Limit (PEL) (15 mg/m^3^) for total TiO_2_ dust [188]. During the past two decades, increasing research on nanotoxicity has led to the derivation of significantly lower exposure limits, mostly for the workplace (Table 3). In 2011, NIOSH recommended a Recommended Exposure Level (REL) for ultrafine (=NPs) TiO_2_ (0.3 mg/m^3^), which is eight times lower than the REL of fine (=MPs) TiO_2_ (2.4 mg/m^3^) [141].

In general, lung inflammation and lung cancer were used as critical effects to set exposure limits. Lung inflammation is often considered a threshold effect [151], where a No Observed Adverse Effect Concentration (NOAEC) is selected as “point of departure” (POD), which is subsequently divided by assessment or uncertainty factors (AFs/UFs) to account for interspecies extrapolation, intraspecies variability, extrapolation from sub-chronic to chronic exposures, the incompleteness of databases, etc. Neutrophil influx is a frequently used dose-dependent marker of pulmonary inflammation [151].

Unlike lung inflammation, cancer is generally considered a non-threshold effect. For such effects, the dose–response curve is only used to set exposure limits at acceptable excess risk levels. This was applied in 2018 by the Danish National Research Centre for the Working Environment (NRCWE), as they could not rule out the possibility of direct DNA damage by TiO_2_ NPs [151]. However, as the current evidence suggests that secondary genotoxicity is the main cause of TiO_2_ NP carcinogenicity, a threshold-based limit for pulmonary inflammation may be appropriate. From this perspective, the chronic OEL and Toxicity Reference Value (TRV) of the French Agence Nationale de sécurité sanitaire, de l’alimentation, de l’environnement et du travail (ANSES) is expected to provide a high level of protection for workers and the general population, respectively [179,180]. The same can be said about the subchronic Acceptable Exposure Limit (AEL) of the Belgian research institute Sciensano [46].

## 7. Exposure Assessment: How Many TiO_2_ (Nano)Particles Are Released?

The quantification of particle release and inhalation during face mask use remains problematic due to a lack of data. While TiO_2_ (nano)particles were released in the order of the detection limit in an experimental set up, mimicking real-life breathing, this direct method failed to provide reliable data [89]. Only indirect leaching and washing experiments have proven to be cheap methods for estimating particle release [88,89,90,91,115,193,194]. These experimental conditions are more intense than real-life breathing with humid air and saliva, providing a conservative, worst-case estimate of potential exposure.

The release of Ti (both NPs and MPs, 0.5–14.4% < 260 nm) from five different textile samples (t-shirts and trousers) was between 0.01 and 0.06 wt% after one washing cycle [115]. One sample (83% polyester, 17% wool) released more Ti (3.4 wt%) due to the late addition of a Si/Ti-AgCl/TiO_2_ nanocomposite in the fabrication process that is weakly bound to the fibre surface [115]. In another study, UV-protected textiles did not release significant amounts of TiO_2_ particles (<450 nm) after 30 min incubation in artificial sweat. A measurable release of both Ti and Ag was reported from one sample (polyester and wool), especially in acidic sweat [193]. In Rovira et al. [194], one polyester textile sample released 1.28 mg Ti/kg (7.1% migration rate) in artificial sweat, while leaching from other textiles generally remained below the detection limit. Sullivan et al. [91] submerged disposable surgical face masks in 250 mL water for 24 h. Ti was not detected in the leachate of four masks, but it ranged between 0.06 and 0.64 µg/l in the leachate of four other masks (corresponding to 0.015–0.16 µg Ti/mask) [91]. In a similar study with surgical and FFP2 masks, the release of TiO_2_ per mask was between 0.001 and 0.002 µg/l water [90].

Recently, extensive research was conducted on TiO_2_ in face masks obtained from suppliers in Belgium and the EU. Agglomerated, near-spherical TiO_2_ particles were detected in different disposable and reusable masks of polyester, polyamide, and bi-component fibres, but not in cotton and meltblown non-woven and some thermobonded non-woven fabrics [46]. Between 6 and 65% of the particles were nano-sized, with median particle sizes ranging from 89 to 184 nm [46]. The total mass of TiO_2_ strongly varied, ranging from 791 to 152,345 µg per mask with from 17 to 4394 µg at the fibre surface [46]. It was assumed that only particles at the fibre surface have the potential to leach [46], as fully polymer-embedded NPs > 5 nm have extremely low migration capacities [195]. In a subsequent study, ten masks were selected for leaching experiments, shaken in artificial sweat. Only one reusable mask released Ti in quantities above the detection limit [88]. The external and internal layers of this reusable mask were made of polyester, polyamide, and elastane. After 1 h, 0.3% (34 ± 7 µg) of the total Ti content leached into the artificial sweat. After 8 h, this increased to 0.4% (47 ± 24 µg). The mask also leached silver, releasing 29% (51 ± 3 µg) and 43% (76 ± 23 µg) of the total silver content after 1 and 8 h, respectively [88].

## 8. Risk Characterisation of Different Exposure Scenarios

As no direct measurements of inhalation exposure from face masks are available, few risk assessments have been carried out to date. A two-step method to screen mask safety was recently proposed by Sciensano [46,88,89]. The first step checks if a mask is safe-by-design. Without assuming the likelihood of particle release, the mass of TiO_2_ on the fibre surface is compared with the AEL (Table 3) calculated for one mask (AEL_mask_ = 3.6 µg ultrafine TiO_2_, assuming the use of two masks, each for 4 h, with a breathing rate of 1.25 m^3^/h during 8 h) [46]. It was found that this AEL_mask_ was exceeded by all 12 masks studied [46]. Subsequently, leaching experiments were performed by Montalvo et al. [88] as a higher-tier approach to assess the safety of masks that were found not safe-by-design. Only one out of ten masks released quantifiable amounts of TiO_2_, strongly exceeding the generic AEL_mask_ [88].

Until a representative experimental set up is developed that directly measures particle release during breathing, a leaching experiment in water and artificial sweat may be used for a conservative exposure estimate. The aforementioned studies have shown that the cumulative TiO_2_ release from masks and textiles after several hours of leaching is typically below the limit of detection or limit of quantification (e.g., 0.16 µg Ti/l in [88]). Hence, no risk is assumed for the vast majority of masks, as real-life exposure will be far below the conservative exposure limits (Table 3). Nevertheless, some masks or textile samples show higher Ti migration rates (up to 7.1% [194]), requiring further evaluation.

It can be expected that a significant part of the released Ti is nano-sized, representing 6–65% of all TiO_2_ in face masks studied by Verleysen et al. [46]. Until precise size measurements of the leached particles are available, risks must be assessed using NP-exposure limits, which are more protective than MP-exposure limits.

Using the highest measurements of Sullivan et al. [91] and Montalvo et al. [88] as proxies to simulate potential Time-Weighted Averages (TWAs) of TiO_2_ inhalation, four (sub)chronic, worst-case exposure scenarios are outlined (Table 4). Scenarios 1 and 3 assume one mask is worn for 8 h/day, while scenarios 2 and 4 simulate more intensive use, with two masks worn consecutively for 4 h/day each (total exposure 8 h/day).

In scenario 1, repeated use of the surgical mask tested by Sullivan et al. [91] poses no health risk. In the more intensive scenario 2, the ANSES and Sciensano threshold-based exposure limits for pulmonary inflammation suggest no risk (Table 4). While the Danish OEL for lung cancer (1:100,000) is slightly exceeded, one should note that this exposure limit holds for continued exposure over 45 years, which is unlikely if exposure occurs through wearing a mask (Table 3). Furthermore, the actual particle release during 8 h of breathing humid air is likely considerably lower than that in the 24 h leaching experiment in water.

According to scenarios 3 and 4, the reusable mask of Montalvo et al. [88], with the highest TiO_2_ concentration in the leachate, poses health risks. While the simulated exposures are below the NIOSH’s REL, they exceed all other limit values, including the chronic OELs of ANSES and NRCWE for workers and the subchronic AEL of Sciensano (Table 4). Of major concern is that the ANSES TRV for the general population is exceeded by 65 and 95 times in scenarios 3 and 4, respectively. Although occasional/single use of these masks presumably has a negligible risk, increased inflammatory effects cannot be ruled out when used daily during longer periods (e.g., pandemics).

Finally, it is important to point out that exposure is not limited to TiO_2_ NPs. As an excessive amount of ionic silver leached from the mask studied in scenarios 3 and 4 [88], the combined effects of simultaneous exposure to silver and TiO_2_ cannot be ruled out a priori. Unfortunately, the Cumulative Risk Assessment (CRA) of different nanomaterials is still in its early stages, rendering further evaluation difficult.

## 9. Discussion

Face masks have proven to be effective tools in preventing the airborne transmission of viruses, significantly reducing the risk of COVID-19 infection. On top of physical filtration, face mask manufacturers increasingly use metal NPs for antimicrobial properties, primarily mediated by ROS production and oxidative stress. These NPs are incorporated into polymer fibres as nanocomposites, or applied as coatings. While the nanoscale of these particles offers them advantages over larger particles (increased reactivity, better dispersion through fibres, etc.), a significant limitation is their tendency to be more toxic to humans.

TiO_2_ NPs are often used in face masks. However, they are considered a possible human carcinogen. It is widely accepted that TiO_2_ NPs cause indirect/secondary genotoxic effects, while some indications exist for direct DNA interactions. While studies in rats demonstrated an excess incidence of both benign and malignant lung tumours after chronic exposure, the evidence for humans is still debated due to interspecies differences, the unrealistically high concentrations administered in the animal experiments, and the lack of solid evidence in epidemiological studies among workers. Moreover, the inhalation of TiO_2_ NPs can induce pulmonary inflammation and cause histopathological changes (e.g., fibrosis), while asthmatic symptoms may worsen. Most exposure limits for TiO_2_ NPs are threshold-based and relate to the pulmonary inflammation observed in rats. Other potential effects are known from rodent studies and in vitro experiments with human cell lines, including adverse immune responses, neurotoxicity, and cardiovascular effects. In addition, offspring may be affected due to the capacity of TiO_2_ NPs to cross the blood–testis and blood–placental barriers. Hence, these effects warrant a comprehensive and precautionary assessment of applications involving TiO_2_ NPs.

The lack of direct exposure data complicates the risk assessment of inhaled exposure to TiO_2_ NPs from face masks. The quantifications of potential particle release are indirect, based on leaching experiments. As the experimental conditions of the latter are more extreme than real-life breathing conditions, leaching data provide a worst-case exposure estimate. While the release is mostly below the detection limit, some textile samples leach measurable Ti amounts. A considerable portion is probably nano-sized, as a recent study found that 6–65% of TiO_2_ in a series of face masks consists of NPs [46]. To assess the risks of masks with high release, four conservative (sub)chronic exposure scenarios were simulated, using published measurements. While most masks are concluded to be safe, especially during occasional/single use, a minority of masks on the EU market seem to be inadequate for prolonged, intensive use. In retrospect, the risk identified from the prolonged use of these TiO_2_-treated masks is small compared with the overall protective benefit of wearing face masks against SARS-CoV-2. Given the initially high crude mortality and case fatality rates, there can be no doubt that the benefits of wearing masks during the pandemic outweighed NP-associated risks. Nevertheless, mask nanosafety should be ensured in the future.

Overall, it can be questioned whether the biocidal applications of metal (nano)particles are needed in face masks. Multiple studies quantified TiO_2,_ silver, zinc, and copper in masks and their leachates. While the biocidal effect of Ag and CuO NPs is mainly mediated through the continuous release of ions, ZnO NPs combine both ion release with photocatalysis. In contrast, TiO_2_ NPs mainly produce ROS by UV-dependent photocatalysis. Therefore, it is doubtful whether nano-TiO_2_ truly achieves the antimicrobial activity claimed by manufacturers (see, e.g., [98]), given that only about 3% of sunlight at the Earth’s surface can be utilised, and this percentage is even lower indoors. Although the photoresponse of TiO_2_ can be extended to visible light through surface modification and doping with (non-)metals, these modifications complicate toxicity assessments. Hence, the biocidal activity of light-independent metals like silver is more reliable. Unfortunately, these may also cause health effects [120,196,197,198,199,200]. While some researchers estimated the risks to be acceptable [45], others concluded that silver-based biocides in face masks also require regulatory control and standardisation [88,89]. The legal framework for these “biocidal” applications was observed to be ambiguous and often non-existent under Regulation (EU) No 528/2012. In addition to the toxicity of metal NPs, this review shows that respirators and surgical masks provide substantial protection against large respiratory droplets through effective physical filtration. Even some cloth masks achieved relatively good results when they included multiple cotton layers of a high weave density. Consequently, the added protective value of metal (nano)particles in face masks appears to be very low for the general population, especially if masks are changed daily. For healthcare workers, further investigation is needed to weigh the advantages and disadvantages, although it is evident that the use of TiO_2_ should be excluded.

## 10. Conclusions

“Antimicrobial” face masks should be subject to stringent quality control measures and require a clear legislative framework addressing their safety, accounting for various uncertainties. The overall benefit of “biocidal” metal (nano)particles in face masks for the general population appears to be very low, especially for TiO_2_ NPs. A major drawback for human risk assessment is that exposure can only be approximated indirectly, using leaching experiments as a proxy for potential particle release. Moreover, the effects of combined exposure to both silver (nano)particles and TiO_2_ NPs remain unknown. The development of reliable strategies for a Cumulative Risk Assessment of nanomaterials is much needed. Therefore, this study concurs with Skojac et al. [63] that TiO_2_ NPs should be used with great care until sufficient human exposure and toxicological data are available, allowing for a more realistic risk assessment. While most masks are safe, especially for occasional/single use, the nanosafety of a minority of face masks on the European market may be inadequate for prolonged and intensive use. Considering the potential safety issues and the limited added protective value of TiO_2_ NPs, it is recommended to ban all applications of TiO_2_ in face masks (both NPs and MPs) based on the precautionary principle.

## Figures and Tables

**Table 2:** A general summary of some key properties related to the antimicrobial activity of metal nanoparticles. A more detailed and nuanced overview is provided in the text.

Properties	Silver	Copper	Zinc	TiO_2_	Remarks
Release of ions	Yes	Yes	Yes	No	Zn^2+^, Ag^+^, Cu^2+^ are released. These ions contribute to cellular disruption, and oxidative stress.
Light-dependent	No	No	Partial	Yes	TiO_2_ requires UV light for ROS generation through photocatalysis, while ZnO also acts by releasing ions.
ROS generation and oxidative stress	Yes	Yes	Yes	Yes	ROS generation and increased oxidative stress are key mechanisms for cytotoxicity and antimicrobial activity.
Disruption of cell membrane/viral envelope	Yes	Yes	Yes	Yes	Direct interaction leads to structural damage, increased permeability, and cellular leakage.
DNA damage	Yes	Yes	Yes	Yes	Primarily indirect genotoxicity by ROS. Conclusions regarding their direct genotoxicity require further study for each NP type.
Protein damage	Yes	Yes	Yes	Yes	Protein damage occurs via ROS or direct interactions with released metal ions (e.g., Ag^+^ binds thiol groups).
*E. coli* cytotoxicity: Predicted log(1/EC_50_)	4.07	3.35	3.39	1.95	Values derived using the nano-QSAR model of Mu et al. [94] for nano- Ag_2_O, CuO, ZnO, and TiO_2_. Higher values indicate higher cytotoxicity and vice versa. Hence, the cytotoxicity of TiO_2_ NPs is relatively weak.

**Table 3:** A selection of health-based exposure limits for the inhalation of TiO_2_ NPs, proposed by different institutes and European projects. REL = Recommended Exposure Limit; INEL = Indicative No-Effect Level; OEL = Occupational Exposure Limit; STEL = Short-Term Exposure Limit; AEL = Acceptable Exposure Level; TWA = Time-Weighted Average; NOAEC = No Observed Adverse Effect Concentration; HEC = Human Equivalent Concentration; AF = Assessment Factor.

Institute or Project	Limit	Value	Exposure Details	Remarks	Source
NIOSH	REL	300 µg/m^3^	Chronic. 10 h/day TWA, 40 h work week.	Reduces the excess human lung cancer risk to below 1:1000. Benchmark dose approach with model averaging, based on chronic rat inhalation studies (e.g., [166]).	[141]
ENRHES EU project	INEL	17 µg/m^3^	Chronic. 8 h/day.	Prevents pulmonary inflammation. Threshold-based. Derived with AF_total_ = 15 from corrected NOAEC (0.25 mg/m^3^) in a sub-chronic rat inhalation study [189].	[190]
Scaffold EU project	OEL	100 µg/m^3^	Chronic. 8 h/day.	Prevents pulmonary inflammation. Threshold-based. Derived with AF_total_ = 2.5 from corrected NOAEC (0.25 mg/m^3^) in a sub-chronic rat inhalation study [189].	[191]
NRCWE	OEL	10 µg/m^3^	Chronic. 8 h/day.	Prevents pulmonary inflammation. Threshold-based. Derived with AF_total_ = 25 from corrected NOAEC (0.25 mg/m^3^) in a sub-chronic rat inhalation study [189].	[151]
	OEL _1:100,000_	0.04 µg/m^3^	Chronic. 8 h/day, 40 h work week, 45 years.	Reduces the excess human lung cancer risk to 1:100,000. Non-threshold based, assuming linear-dose–response. Based on estimated human lung burden, derived from chronic rat inhalation study [166] and pulmonary deposition fraction in mice [192].	[151]
ANSES	TRV	0.12 µg/m^3^	Chronic (general population)	Prevents pulmonary inflammation. Threshold-based. Derived with AF_total_ = 225 from corrected NOAEC_HEC_ (0.028 mg/m^3^) in a sub-chronic rat inhalation study [189]. Applicable to Aeroxide TiO_2_ P25 (80% anatase/20% rutile; 21 nm).	[179]
	OEL	0.80 µg/m^3^	Chronic. 8 h/day TWA, 240 days/year, life-long.	Prevents pulmonary inflammation. Threshold-based. Derived with AF_total_ = 81 from corrected NOAEC_HEC_ (0.065 mg/m^3^) in a sub-chronic rat inhalation study [189]. Applicable to Aeroxide TiO_2_ P25 (80% anatase/20% rutile; 21 nm).	[180]
	STEL	4 µg/m^3^	15 min TWA.	Threshold-based. Maximum 5× 8 h OEL.	[180]
Sciensano	AEL	0.72 µg/m^3^	Subchronic. 8 h/day.	Prevents pulmonary inflammation. Threshold-based. Derived with AF_total_ = 90 from corrected NOAEC_HEC_ (0.065 mg/m^3^) in a sub-chronic rat inhalation study [189]. Applicable to Aeroxide TiO_2_ P25 (80% anatase/20% rutile; 21 nm).	[46]

**Table 4:** Conservative risk assessment of four theoretical, long-term, (sub)chronic exposure scenarios, assuming that the daily inhaled amount of TiO_2_ equals the measurements from two leaching studies [88,91]. All exposure limits consider TiO_2_ NPs. Bold RCRs are >1. RCR = Risk Characterisation Ratio; TWA = Time-Weighted Average = (C_1_T_1_ + C_2_T_2_ +…+ C_n_T_n_)/(T_1_ + T_2_ +…+ T_n_). The conversion of Ti to TiO_2_ mass uses a multiplication factor of 1.668.

Theoretical Exposure Scenario	Ti (µg) Leached from Mask	Converted to TiO_2_ (µg)	Simulated TWA TiO_2_ Inhalation (µg/m^3^)	Exposure Limit	RCR
*Scenario 1:* Adult wearing 1 × face mask 2 of Sullivan et al. [91] for 8 h/day; air inhalation rate 1.25 m^3^/h. Assumption: inhaled TiO_2_ during 8 h equals the measured amount of TiO_2_ in water leachate (=0.64 µg Ti/L × 0.25 L/mask × 1.668) after a contact time of 24 h.	0.16	0.27	0.027	NIOSH—REL (300 µg/m^3^)	8.9 × 10^−5^
				NRCWE—OEL _1:100,000_ (0.04 µg/m^3^)	0.67
				ANSES—OEL (0.8 µg/m^3^)	0.03
				Sciensano—AEL (0.72 µg/m^3^)	0.04
				ANSES—TRV (0.12 µg/m^3^)	0.22
*Scenario 2:* Adult wearing 2 × face mask 2 of Sullivan et al. [91]; each mask worn for 4 h/day; air inhalation rate 1.25 m^3^/h. Assumption: inhaled TiO_2_ during 8 h equals 2 × the measured amount of TiO_2_ in water leachate after a contact time of 24 h.	0.32	0.53	0.053	NIOSH—REL (300 µg/m^3^)	1.8 × 10^−4^
				NRCWE—OEL _1:100,000_ (0.04 µg/m^3^)	**1.33**
				ANSES—OEL (0.8 µg/m^3^)	0.07
				Sciensano—AEL (0.72 µg/m^3^)	0.07
				ANSES—TRV (0.12 µg/m^3^)	0.44
*Scenario 3:* Adult wearing 1 × AgMask18 of Montalvo et al. [88] for 8 h/day; inhalation rate 1.25 m^3^/h. Assumption: inhaled TiO_2_ during 8 h equals the measured amount of TiO_2_ in artificial sweat leachate after a contact time of 8 h (=47 µg Ti/mask × 1.668).	47	78.40	7.840	NIOSH—REL (300 µg/m^3^)	0.03
				NRCWE—OEL _1:100,000_ (0.04 µg/m^3^)	**195.99**
				ANSES—OEL (0.8 µg/m^3^)	**9.80**
				Sciensano—AEL (0.72 µg/m^3^)	**10.89**
				ANSES—TRV (0.12 µg/m^3^)	**65.33**
*Scenario 4:* Adult wearing 2 × AgMask18 of Montalvo et al. [88]; each mask worn for 4 h/day; air inhalation rate 1.25 m^3^/h. Assumption: inhaled TiO_2_ during 8 h equals 2 × the measured amount of TiO_2_ in artificial sweat leachate after a contact time of 1 h (=2 masks × 34 µg Ti/mask × 1.668).	68	113.42	11.342	NIOSH—REL (300 µg/m^3^)	0.04
				NRCWE—OEL _1:100,000_ (0.04 µg/m^3^)	**283.56**
				ANSES—OEL (0.8 µg/m^3^)	**14.18**
				Sciensano—AEL (0.72 µg/m^3^)	**15.75**
				ANSES—TRV (0.12 µg/m^3^)	**94.52**

## Data Availability

Not applicable. No new data were created in this study.

## Abbreviations

The following abbreviations are used in this manuscript:

AEL	Acceptable exposure limit
AEL_mask_	Acceptable exposure limit for one face mask
AF	Assessment factor
ANSES	Agence nationale de sécurité sanitaire, de l’ alimentation, de l’ environnement et du travail
AOP	Adverse outcome pathway
aOR	Adjusted odds ratio
ASTM	American Society for Testing and Materials
ATP	Adenosine triphosphate
BFE	Bacterial filtration efficiency
bw	Body weight
CB	Conduction band
CFM	Cubic feet per minute
CI	Confidence Interval
COVID-19	Coronavirus disease 2019
CRA	Cumulative risk assessment
DNA	Deoxyribonucleic acid
EC_50_	Half maximal effective concentration
ECHA	European Chemicals Agency
EFSA	European Food Safety Authority
*E_g_*	Energy band gap
EN	Europäische Norm
ENRHES	Engineered Nanoparticles: Review of Health and Environmental Safety
RNA	Ribonucleic acid
EPA	Environmental Protection Agency
FFP	Filtering facepiece
HEC	Human equivalent concentration
IARC	International Agency for Research on Cancer
INEL	Indicative no-effect level
MP	Microparticle (fine particle > 100 nm)
NIOSH	National Institute for Occupational Safety and Health
NOAEC	No Observed Adverse Effect Concentration
NP	Nanoparticle (ultrafine particle, <100 nm)
NRCWE	National Research Centre for the Working Environment
OEL	Occupational Exposure Limit
OPE	Organophosphorus ester
OPFR	Organophosphate flame retardant
OSHA	Occupational Safety and Health Administration
PAH	Polycyclic aromatic hydrocarbon
PEL	Permissible exposure limit
PFAS	Per- and polyfluoroalkyl substances
POD	Point of departure
PPE	Personal protective equipment
PSLT	Poorly soluble low toxicity
QSAR	Quantitative structure-activity relationship
RAC	Committee for risk assessment
ROS	Reactive oxygen species
RR	Relative risk
SHC	Superior Health Council of Belgium
SMR	Standardised mortality ratio
STEL	Short-term exposure limit
TiO_2_	Titanium dioxide
TWA	Time-weighted average
TPI	Threads per inch
TRV	Toxicity reference value
UF	Uncertainty factor
